# Radiation field size and dose determine oncologic outcome in esophageal cancer

**DOI:** 10.1186/s12957-016-1024-0

**Published:** 2016-10-13

**Authors:** Cengiz Gemici, Gokhan Yaprak, Hasan Fevzi Batirel, Mahmut Ilhan, Alpaslan Mayadagli

**Affiliations:** 1 Department of Radiation Oncology, Dr. Lutfi Kirdar Kartal Education and Research Hospital, Cevizli, Istanbul, Turkey; 2 Department of Thoracic Surgery, Marmara University Medical Faculty, Istanbul, Turkey; 3 Department of Medical Oncology, Avrasya Hospital, Istanbul, Turkey; 4 Department of Radiation Oncology, Bezmialem Vakif University Medical Faculty, Istanbul, Turkey

**Keywords:** Esophageal cancer, Concomitant chemoradiotherapy, Locoregional recurrence, Overall survival

## Abstract

**Background:**

Locoregional recurrence is a major problem in esophageal cancer patients treated with definitive concomitant chemoradiotherapy. Approximately half of the patients fail locoregionally. We analyzed the impact of enlarged radiation field size and higher radiation dose incorporated to chemoradiotherapy on oncologic outcome.

**Methods:**

Seventy-four consecutive patients with histologically proven nonmetastatic squamous or adenocarcinoma of the esophagus were included in this retrospective analysis. All patients were locally advanced cT3–T4 and/or cN0-1. Treatment consisted of either definitive concomitant chemoradiotherapy (Def-CRT) (*n* = 49, 66 %) or preoperative concomitant chemoradiotherapy (Pre-CRT) followed by surgical resection (*n* = 25, 34 %). Patients were treated with longer radiation fields. Clinical target volume (CTV) was obtained by giving 8–10 cm margins to the craniocaudal borders of gross tumor volume (GTV) instead of 4–5 cm globally accepted margins, and some patients in Def-CRT group received radiation doses higher than 50 Gy.

**Results:**

Isolated locoregional recurrences were observed in 9 out of 49 patients (18 %) in the Def-CRT group and in 1 out of 25 patients (3.8 %) in the Pre-CRT group (*p* = 0.15). The 5-year survival rate was 59 % in the Def-CRT group and 50 % in the Pre-CRT group (*p* = 0.72). Radiation dose was important in the Def-CRT group. Patients treated with >50 Gy (11 out of 49 patients) had better survival with respect to patients treated with 50 Gy (38 out of 49 patients). Five-year survivals were 91 and 50 %, respectively (*p* = 0.013).

**Conclusions:**

Radiation treatment planning by enlarged radiation fields in esophageal cancer decreases locoregional recurrences considerably with respect to the results reported in the literature by standard radiation fields (18 vs >50 %). Radiation dose is as important as radiation field size; patients in the Def-CRT group treated with ≥50 Gy had better survival in comparison to patients treated with 50 Gy.

## Background

Locoregional recurrence is a major concern and the primary mode of failure in esophageal cancer patients treated either with surgery or definitive chemoradiotherapy. The unique lymphatic network of the esophagus and the absence of serosal covering around the organ are the two major causes of high locoregional failures after treatment [1–5].

Extensive, longitudinal interconnecting system of lymphatics facilitates not only early lympathic spread of the tumors but also potential risk for lymphatic involvement longitudinally throughout the entire length of the organ rather than the segmental involvement of nodal areas [5]. Metastases to anatomically distant lymph nodes could develop even in the early phases of lymphatic invasion and up to 8 cm or more of normal tissue can exist between the gross tumor and its micrometastases [5–10].

Lymph node metastases can be observed even with superficial esophageal tumors. While the reported incidence of nodal involvement is around 14 to 21 % for T1 tumors, this chiffre rises immediately up to 60 % for T2 tumors [5–10]. Autopsy findings demonstrate residual or recurrent tumor in 60 % of the patients after curative surgery. While local recurrences were observed in 25.6 % of autopsied cases, lymph node metastases were observed in 41.9 % of the cases [11].

Anatomic difficulty to remove the tumor and the lymph nodes completely is suggested for high locoregional failures after surgical resection [11].

One striking and clinically unknown detail about the involved lymph nodes in esophageal cancer is their microscopic involvement in majority of the cases. Most of the nodal involvement would not be radiologically or clinically identifiable. The involved lymph nodes are clinically evident in only 1/3rd of the cases [8–10]. Therefore, it is not appropriate to rely on varying imaging modalities to define areas of spread of the disease. Spread pattern of lymph node metastases in defining target volumes will be more valuable in radiotherapy planning and field design.

Locoregional failures with curative intent surgery in contemporary trials range from 32 to 45 % [2, 11–14]. Locoregional failures are also the major pattern of failure in patients treated with definitive chemoradiotherapy (Def-CRT), at least half of the patients fail locoregionally [3, 4, 15]. Undetected persistent or recurrent subclinical locoregional disease is very common in curatively treated patients, and there are very high discordance rates between clinic and pathologic stages in these patients [11, 16].

There is universal consensus in radiation therapy target volume delineation and radiation therapy target doses in esophageal cancer treatment. The radiation field design and dose of the Intergroup 0123 Trial became the worldwide gold standard in definitive treatment and recently, even in smaller radiation fields and lower radiation doses in preoperative treatment of esophageal cancer [4, 15, 17–20]. In the Intergroup 0123 Trial, cranial and caudal borders of the radiation fields were 5 cm beyond the gross tumor volume (GTV); the lateral, anterior, and posterior borders of the fields were 2 cm beyond the GTV. Although supraclavicular lymphatics were included for tumors of the cervical esophagus, no special attention was given to include the regional lymphatics beyond 5 cm cranial and caudal borders of GTV in other tumor locations [4].

The aim of this study is to analyze our patient experience with locally advanced esophageal carcinoma who underwent either surgery following preoperative chemoradiotherapy (Pre-CRT) or Def-CRT using longer radiation fields at craniocaudal borders of GTV and higher radiation doses and the outcome of these treatment parameters in terms of locoregional recurrence and survival.

## Methods

Seventy-four consecutive patients with histologically proven non-metastatic squamous or adenocarcinoma of the cervical, upper, middle, and lower third of the esophagus were included in this retrospective study. Three-hundred twenty esophageal cancer patients were evaluated at the Dr. Lutfi Kirdar Kartal Education and Research Hospital during this period. Two-hundred forty-four patients were excluded from multimodality treatment mainly due to patients undergoing straightforward surgery, exclusion secondary to patient-related factors (prohibitive comorbidities for multimodality treatment) or metastatic disease at presentation. All patients had clinical T3–4, N0–1, and M0 tumors according to the sixth edition of American Joint Commission on Cancer staging manual [21]. Tumor stage was evaluated by physical examination, neck, thoracic, and abdominal computerized tomography (CT), upper gastrointestinal endoscopy, and, after 2008, by fluorodeoxyglucose positron emission tomography (PET-CT) (*n* = 27, 36 %). The patients were analyzed in two groups. The first group comprised patients seen first by a radiation oncologist and treated with Def-CRT (*n* = 49, 66 %) with no planned esophagectomy, and the second group comprised patients seen first by a thoracic surgeon and referred for Pre-CRT (*n* = 25, 34 %).

Patient and tumor characteristics are summarized in Table 1. There were no differences between the two groups with respect to patient and tumor characteristics.

**Table 1:** Patient and tumor characteristics

Patient characteristics	Pre-CRT (*n* = 25)		Def-CRT (*n* = 49)		*P* value
	*N*	(%)	*n*	(%)	
Age, years					
Median	53 ± 10.7		58.6 ± 12.1		0.055
Range	32–67		30–82		
Gender (female/male)	17/8		28/21		0.37
Clinical T stage					
3	13		29		0.56
4	12		20		
Nodal stage					
0	8		17		0.82
1	17		32		
Histology					
Adenocarcinoma	2		6		0.58
Squamous cell carcinoma	23		43		
Esophageal location					
Cervical	0		7		0.15
Upper	0		4		
Middle	13		18		
Lower	12		20		

*CRT* chemoradiotherapy

Radiotherapy was administered using two-dimensional (2D) planning technique (*n* = 30) and three-dimensional (3D) conformal planning (*n* = 44) after 2007. Mega-voltage photon energy ≥6 MV was used. While anterior and posterior parallel opposed fields were used up to a total dose of 46 Gy in 23 fractions with 2 Gy fractions per day, 5 days per week in patients treated with 2D radiation planning; anterior, posterior, and two lateral or posterior oblique fields with same dose and fractionation were used in patients treated with 3D radiation planning. While radiation dose was 46 Gy in the Pre-CRT group (*n* = 25), it was 50 Gy for middle and lower thoracic tumors (*n* = 38) and between 50.1 and 60 Gy for upper thoracic and cervical esophageal tumors (*n* = 11) in the Def-CRT group.

The GTV was determined by computed tomography, barium swallow, and endoscopy with or without PET-CT. Initial clinical target volume (CTV1) was obtained by expanding the GTV longitudinally at cranial and caudal margins by 8–10 cm and transversal margins of 1.5–2 cm. The planning target volume (PTV) contained the CTV1 and additional margins of 0.5 cm in all directions for consideration of organ movements. After 46 Gy, boost was planned for patients treated with Def-CRT. CTV2 for boost was obtained by expanding GTV longitudinally by 4–5 cm and transversally by 1 cm. Two lateral fields were used for patients treated with 2D planning (*n* = 20) and one anterior (and or posterior) and or two lateral or posterior oblique fields were used for patients treated with 3D planning (*n* = 29).

Special attention has been given to keep the spinal cord, heart, and lung radiation doses at tolerance levels, and this has been achieved either by blocking for patients treated by 2D radiation planning or by multileaf collimators treated by 3D radiation planning.

Chemotherapy was started on the same day as radiotherapy. Several chemotherapy schemata either weekly, three weekly, or monthly cycles were administered during the study. The most common chemotherapy combination used was cisplatin (CDDP) weekly either alone (40 mg/m^2^) or together with other chemotherapeutic agents (*n* = 56) and CDDP with infusional 5-fluorouracil (5-FU) (*n* = 15) (CDDP, 75 mg/m^2^ on the first day of weeks 1 and 5 of radiotherapy and 5-FU 1 g/m^2^ for the first 4 days of weeks 1 and 5 of radiotherapy). Table 2 summarizes the treatment characteristics. Chemotherapy was not continued after Def-CRT or Pre-CRT as adjuvant treatment.

**Table 2:** Treatment characteristics

Treatment characteristics	Pre-CRT (*n* = 25)		Def-CRT group (*n* = 49)		*P* value
	*n*	(%)	*n*	(%)	
Radiation planning					
Conventional (2D)	10	40	20	41	0.95
Conformal (3D)	15	60	29	59	
Chemotherapy regimes					
Cisplatin + infusional 5-FU	5	20	10	20	0.7
Weekly cisplatin	7	28	11	22	
Weekly cisplatin + oral UFT	8	32	16	33	
Weekly cisplatin + paclitaxel	3	12	7	14	
Paclitaxel + 5-FU	1	4	2	5	
Weekly cisplatin + capecitabine	1	4	3	6	
Radiation dose (cGy)					
4000–5000	25	100	39	78	0.01
5001–6000	0	0	11	22	

*CRT* chemoradiotherapy, *UFT* oral uracil + tegafur

Twenty-five patients referred for Pre-CRT underwent surgery by an experienced thoracic surgeon at a high volume center. Surgery was performed at least 1 month after the end of Pre-CRT. Transthoracic esophagectomy with two-field lymphadenectomy was performed. Salvage surgery was possible in 2 out of 9 patients treated with Def-CRT after isolated locoregional recurrence.

The chemoradiation part of the treatment was performed at Dr. Lutfi Kirdar Kartal Education and Research Hospital, while the esophagectomies were performed at two centers.

Patients were seen 1 month after the end of treatment and every 3 months up to 2 years, every 6 months up to 5 years, and yearly afterwards. Acute and late adverse reactions were evaluated according to the National Cancer Institute’s Common Terminology Criteria for Adverse Events (CTCAE), version 2.0. Late complications were recorded for the lung, heart, and esophagus.

Recurrences were classified as locoregional only, distant only, and synchronous locoregional + distant. Locoregional recurrences (LR) were defined as recurrences at the site of the primary tumor or locoregional lymph nodes. Lymph node recurrences at the celiac trunk or in the supraclavicular region were also considered to be locoregional. Distant recurrences were defined as non-regional lymph node recurrences and systemic metastases. Recurrences were detected by CT scan of the neck, thorax, and abdomen and by endoscopy and/or by PET-CT. LRs were analyzed in relation to the initial PTV and were classified as in-field, when relapse was within PTV, borderline when adjacent to PTV, or out-field when relapse was outside PTV (Table 3).

**Table 3:** Tumor recurrences in relation to initial PTV

Recurrence	Pre-CRT group (*n* = 25)			Def-CRT group (*n* = 49)			*p* value
	Infield	Outfield	Borderline	Infield	Outfield	Borderline	
LRR only	0	0	1	7	1	1	0.15
Distant only	0	4	0	0	5	0	0.25
LRR plus distant	0	3	0	3	1	0	0.98
Total	8			18			0.69

*LRR* locoregional recurrence, *CRT* chemoradiotherapy

The study was approved by local ethical committee of Dr. Lutfi Kirdar Kartal Education and Research Hospital. All patients gave informed consent.

Statistical analysis was performed using Fisher’s exact and Student’s *t* test, chi-square test, Kaplan-Meier survival analysis, and log-rank test for comparisons using IBM SPSS 20.0 software.

## Results

Mean age of the whole cohort was 56.7 ± 11.9 (30–82, 45 females). Median and 5-year survivals were 91 months and 56 %, respectively. Patient and tumor characteristics which underwent both types of treatments are shown in Table 1. The mean follow-up time was 60 months (range 1 to 156 months).

**Fig. 1:**
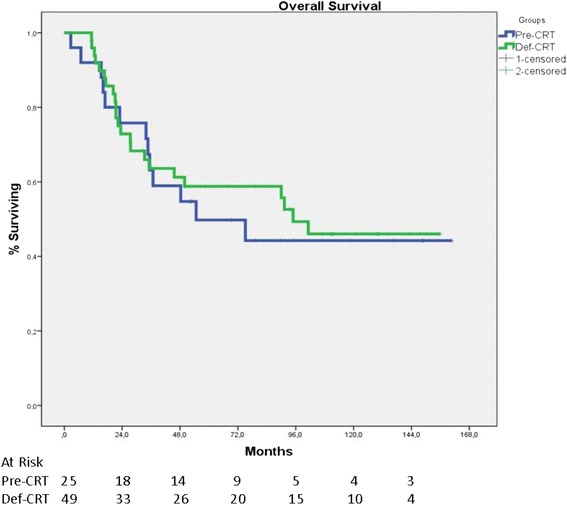
Overall survival of groups who had preoperative chemoradiotherapy (Pre-CRT) or definitive chemoradiotherapy (Def-CRT). Overall median survivals were 55 months (95 % CI 2–108) and 94.9 months and 5-year survival rates were 50 % (95 % CI 40–60) and 59 % (95 % CI 52–66), respectively (*p* = 0.72)

Overall median and 5-year survivals were 95 months and 59 % in the Def-CRT group and 55 months and 50 % in the Pre-CRT group, respectively (*p* = 0.72, Fig. 1). Cancer-specific 5-year survivals were 68 and 61 %, respectively (*p* = 0.77). Cancer-specific median survivals were not reached in both groups.

Locoregional recurrence rate was 18 % in the Def-CRT group and 3.8 % in the Pre-CRT group. The difference between the groups was not statistically different (*p* = 0.15).

The decrease in locoregional recurrences reflected itself in both decreased distant metastases rates and increased 5-year survival rates. Distant metastases were observed in 5 out of 49 patients (10 %) in the Def-CRT group and in 4 out of 25 patients (16 %) in the Pre-CRT group (*p* = 0.25). All recurrences including locoregional, distant, and synchronous locoregional + distant recurrences were observed in 18 out of 49 patients in the Def-CRT group and in 8 out of 25 patients in the Pre-CRT group, 37 vs 32 %, respectively (*p* = 0.69).

Recurrence patterns are summarized in Table 3 and detailed in relation to the initial PTV.

**Fig. 2:**
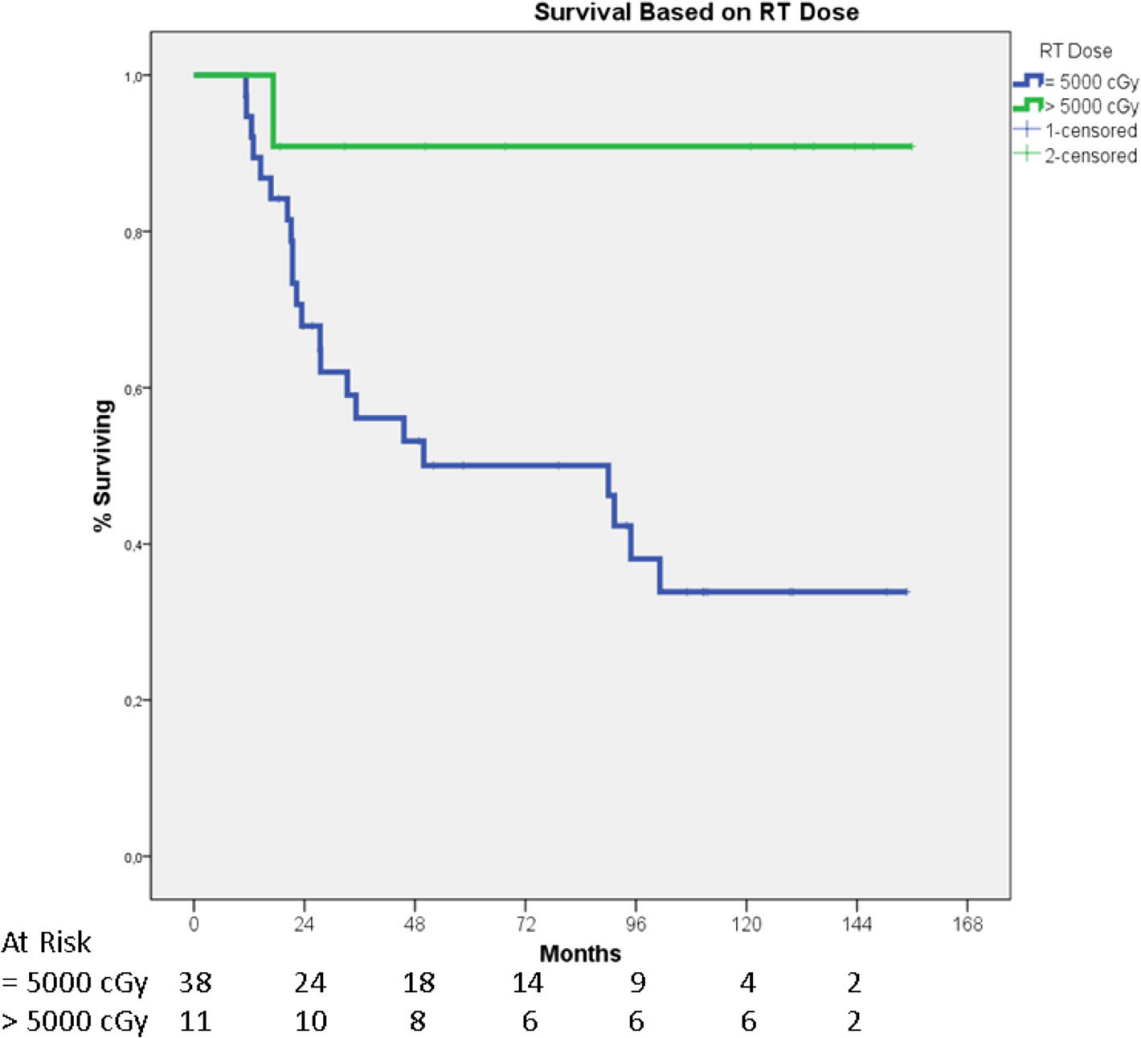
Overall survival of patients who underwent definitive chemoradiotherapy (Def-CRT) with different radiotherapy doses. Overall median survival in patients who received 50 Gy was 90 months (95 % CI 21–159). Median survival was not reached in patients who received >50 Gy. Five-year survival rates were 50 % (95 % CI 42–58) and 91 % (95 % CI 82–100), respectively (*p* = 0.013)

Patients in the Def-CRT group received two different radiation doses. The patients with cervical and upper thoracic esophageal tumors (*n* = 11) were treated with 50.1–60 Gy, and the patients with middle and lower thoracic esophageal tumors were treated with 50 Gy (*n* = 38). While 5-year survival of the patients who were treated with 50 Gy was 50 %, it was 91 % in patients treated with doses between 50.1 and 60 Gy, and the difference was statistically significant (*p* = 0.013) (Fig. 2).

Postoperative morbidity was seen in six patients and included pneumonia and anastomotic leakage. Postoperative mortality was observed within 30 days in 2 patients out of 25 (7 %) due to pneumonia and sepsis. Tumor specimens were carefully analyzed for pathologic complete response, and it was obtained in 8 out of 25 patients (31 %).

Common acute and late adverse events occurring during Pre-CRT and Def-CRT are summarized in Table 4. Major acute adverse events were related to myelosuppression and esophagitis. Grade 3 or higher acute major toxicities were observed in 57 % (*n* = 42) of patients, which necessitated either dose reduction or termination of chemotherapy or a break in the treatment. Grade 3 or higher late adverse events were observed in 39 % (*n* = 29) of patients. There were no acute adverse event-related deaths. While grade 3–4 acute adverse event rates in the Def-CRT and Pre-CRT groups were similar (59 vs 52 %, *p* = 0.55), grade 3–4 late adverse event rates were more common in the Pre-CRT group in comparison to the Def-CRT group (68 vs 24 %, *p* = 0.001). Among late adverse events, esophageal strictures were the most common one and intervention with dilatation was necessary in most of the patients of the Pre-CRT group. This was related to the site of the anastomosis which was left cervical area in operated patients.

**Table 4:** Acute and late adverse events due to treatment

		Pre-CRT		Def-CRT		*p* value
		G3	G4	G3	G4	
Acute toxicity	Esophagitis	3	2	10	3	0.66
	Leukopenia	3	1	5	1	0.72
	Neutropenia	3	1	3	–	0.21
	Anemia	2	1	7	2	0.74
	Thrombocytopenia	2	–	4	1	1
	Nausea	4	–	3	–	0.21
	Vomiting	1	–	6	–	0.41
	Febrile neutropenia	1	–	2	–	1
	Total events	*n* = 8	*n* = 5	*n* = 24	*n* = 5	0.55
Late toxicity	Cardiac	3	–	4	–	0.68
	Pulmonary	4	–	2	–	0.17
	Esophageal (stricture)	12	1	6	–	<0.001
	Total events	*n* = 16	*n* = 1	*n* = 12	*n* = 0	<0.001

## Discussion

Def-CRT (cisplatin, 5-FU, and 50.4 Gy) became the standard non-surgical treatment of patients with locoregionally advanced esophageal cancer after demonstration of survival benefit in the Radiation Therapy Oncology Group (RTOG) 85-01 Trial [3, 22]. However, locoregional recurrence was very high (52 %) and it was the primary mode of failure [3, 22]. The high locoregional failures led to several subsequent trials with the aim of decreasing locoregional recurrences, searching role of either induction chemotherapy (5-FU, cisplatin, Intergroup 0122 Trial), or increasing radiation dose (5-FU, cisplatin, and 64.8 Gy, RTOG 94-05 Trial) [4, 23] or integration of new generation chemotherapeutics, like paclitaxel (RTOG 0113 Trial) [20]. None of those strategies resulted in better locoregional control rates or survival. On the contrary, these approaches resulted in increased toxicity and treatment-related mortality [4, 20, 23]. The radiation treatment parameters (radiation dose and field design) of these new generation trials were the same as that of the Intergroup 0123 Trial.

In our study, radiation treatment planning by lengthening radiation field sizes at craniocaudal margins of GTV decreased locoregional recurrences to 18 % in the Def-CRT and to 3.8 % in the Pre-CRT group (Table 3). This rate is very low when compared with the Intergroup 0123 Trial (>50 %). The decrease in locoregional recurrences translated in to decreased distant metastases rates and increased 5-year survival rates, 59 % in definitively treated and 50 % in preoperatively treated patients, respectively. This is the result of effective coverage and treatment of involved microscopic lymphatic and submucosal spread of the tumor, which was unidentifiable with the current clinical staging.

Several authors tried to incorporate FDG-PET data to improve the accuracy of tumor delineation in radiotherapy planning [24–27]. However, even with the integration of PET data, accuracy of clinical staging is still far away from pathologic staging; there are still problems to support the use of FDG-PET in tumor delineation process for radiation planning [24–27].

Muijs et al. evaluated pathologically the residual tumor presence in relation to radiation therapy target volume after Pre-CRT in 63 resectable esophageal cancer patients [28]. They demonstrated that macroscopically evident residual tumor within CTV and microscopic tumor outside CTV were found in a substantial proportion of the patients, 30 and 14 %, respectively. Residual tumor outside the confines of CTV was found to be an adverse prognostic factor for both locoregional recurrence and survival [28].

Surgery decreases locoregional recurrences in locally advanced esophageal cancer following chemoradiotherapy by removing the residual tumor either in the lymph nodes or in the primary tumor area (within CTV) resting viable due to insufficient radiation dose and also removal of the residual tumor left outside the confines of CTV due to insufficient radiation field size as demonstrated by Muijs et al. [15, 17, 18, 28–36]. Decreased locoregional recurrences in resectable patients following Pre-CRT, on the other hand, result probably from sterilization of tumor either in the lymph nodes or in the primary tumor area which are not accessible to surgical resection due to proximity of the tumor to vital structures (circumferential resection margins) and anatomical difficulty to remove the involved lymph nodes by surgery and in turn increased R0 resection rates provided by decreased tumor bulk after Pre-CRT [15, 17, 18, 28–36]. In both scenarios, surgery compensates for the insufficiency of chemoradiotherapy in sterilizing tumor locoregionally.

Muijs et al. in their abovementioned trial demonstrated two important findings. The first one was related to the insufficiency of radiation dose utilized nowadays in Pre-CRT, 41.4 Gy in 1.8 Gy fractions. This dose is preferentially chosen so low in order to decrease the post chemoradiotherapy surgical complications [18, 28]. But 30 % of the patients had macroscopically evident residual tumor within CTV after 41.4 Gy [28]. Residual tumor within radiation therapy target volume after Def-CRT with the current standard radiation treatment parameters (50.4Gy, 5 cm margins to GTV) is also the most common cause of locoregional failures [3, 4, 32, 33]. Residual tumor besides causing locoregional failure also results in distant metastases and worsens prognosis [28, 32–35]. Residual tumor outside CTV borders were identified in 14 % of the patients’ tumor pieces operated after Pre-CRT with a CTV obtained by adding 3.5 cm margins to GTV craniocaudally [28]. Pre-CRT with this field design and dose still improved the results with respect to surgery alone and became the new treatment standard in resectable esophageal cancer (CROSS Trial) [17, 18]. Surgery provided R0 resection of residual gross tumor within CTV in 30 % of the patients and residual tumor outside the CTV in 14 % of the patients (approximately half of the patients) [28]. Thus, without surgery, almost half of these patients would have failures demonstrating clearly the insufficiency of the radiation treatment parameters (radiation dose and volume) [36]. But in two randomized trials comparing chemoradiotherapy with or without surgery, the survival difference did not reach statistical significance. However, in these two randomized trials, the radiation doses of chemoradiotherapy without surgery groups were higher than those of the preoperative or standard chemoradiotherapy alone trials [15, 37]. Increasing radiation dose of definitive chemoradiotherapy treatment as in these two randomized trials may decrease locoregional recurrences, and surgery may not provide any additional benefit in these patients when radiation doses are optimal.

Button et al. evaluated recurrences clinically by CT and endoscopy in 145 patients in relation to radiation therapy target volumes after Def-CRT with similar radiation field design and dose as in the Intergroup 0123 Study [38]. They reported 49 % local failure rate as in the Intergroup 0123 Trial. The authors considered the radiation fields sufficient since most of the recurrences are identified clinically within radiation therapy target volumes [38]. They suggested that the radiation fields used worldwide were acceptable and with larger radiation fields, the results would not be better. However, pathologic evaluation of recurrences in relation to radiation therapy target volumes by Muijs et al. shed more light on the relation between radiation field size and locoregional recurrences. Muijs et al. demonstrated further that residual tumor presence outside the borders of CTV is associated with worse overall survival. However, the authors instead of emphasizing the importance of inadequate CTV margins in radiation therapy target volume delineation consider the presence of residual tumor cells outside CTV as an indicator of biologically more aggressive tumor behavior [28, 36]. We think that residual tumor presence beyond the confines of CTV results from insufficient radiation field sizes rather than aggressive tumor phenotype as opposed to author’s suggestion [36]. Aggressive tumor phenotype may indirectly result from accelerated repopulation of these untreated residual tumor cells either outside or within CTV, which in turn may confer resistance to eradication of the gross tumor. These cells may constitute a focus for distant dissemination. The tumor cells left outside CTV, besides being untreated by radiation part of the treatment, may not be sterilized efficiently by the chemotherapy part of the treatment. Muijs et al. demonstrated residual microscopic tumor outside CTV in 14 % of the patients after surgery which remained viable despite paclitaxel and carboplatin combination chemotherapy administered weekly throughout the radiation treatment [28]. One can argue that chemotherapy administered during concomitant treatment may not be as efficient as either induction or adjuvant chemotherapy due to low dose intensity. However, neither induction nor adjuvant chemotherapy has shown to decrease the systemic dissemination in esophageal cancer on contrary to common belief among medical oncologists that early treatment and sterilization of subclinical and micrometastatic disease is possible either with induction or adjuvant chemotherapy [39–42]. The main contribution of chemotherapy in esophageal cancer (less with induction, more with concomitant approach) is the result of decrease in tumor bulk with increased resectability and R0 resection rates by surgery rather than sterilization of micrometastatic disease or subclinical disease. In order for the chemotherapy to be effective in patients with esophageal carcinoma both systemically and locoregionally, it should be administered concomitantly with radiotherapy ideally covering the whole tumor cells within the esophagus [36, 42].

The radiation doses for preoperative or adjuvant treatments in solid tumors are generally 46 to 50 Gy, and radiation doses for definitive treatments are generally 60 Gy or higher. However, higher radiation dose, 64.8 Gy, compared with the standard radiation dose, 50.4 Gy, in definitive treatment of locally advanced esophageal cancer in the Intergroup 0123 Trial was not found to be beneficial [4]. There were more treatment-related deaths with 64.8 Gy and similar locoregional recurrences in the two arms of the trial, 56 vs 52 %. Failure of higher radiation dose in the study is probably related to insufficiency of radiation field size leaving residual tumor cells outside, rather than the inefficiency of higher radiation dose. Although there is evidence in the literature regarding the importance of radiation dose escalation on locoregional control rates in esophageal cancer, worldwide accepted dose of definitive treatment is still 50.4 Gy, set as the reference dose as in target volumes after the Intergroup 0123 Trial [4, 43–46]. Radiation dose like longer radiation fields was also found to be an important determinant of outcome in the Def-CRT group of this retrospective study. Patients treated with doses higher than 50 Gy (*n* = 11) had better survival with respect to patients treated with 50 Gy (*n* = 38). Five-year survivals were 92 and 50 %, respectively (*p* = 0.013).

There are several limitations of this study. It is retrospective and various chemotherapy regimens were used over a decade. Our cohort was closely followed and has an unusually high survival rate compared with other contemporary series. This is most likely secondary to selection bias. On the other hand, this strategy may well lead to the elimination of subclinical disease which is very frequent and clinically difficult to detect in esophageal cancer. Our high locoregional control rates even in the Def-CRT group may be related to the elimination of satellite tumorlets and good coverage of subclinical disease within CTV. Surgery can be questioned either in resectable or in locally advanced patients in case the patients are treated with optimal radiation treatment parameters. It is very important to preserve organ while treating efficiently the tumor in the esophagus. It may be possible to achieve similar or better survivals with Def-CRT both in resectable and locally advanced esophageal cancer as in our study.

In conclusion, our results although retrospective are encouraging. There is no major progress in treatment of esophageal cancer since the Intergroup 0123 Trial in unresectable disease and integration of Pre-CRT in resectable disease. Radiation treatment parameters as used in our study should be investigated in randomized trials in order to ameliorate the results and decrease the necessity of surgery in this dismal disease with preservation of organ and better quality of life.

## Conclusions

Radiotherapy is an important treatment modality in esophageal cancer, and it is the standard treatment approach in patients with locally advanced disease. However, radiation treatment parameters mainly radiation field sizes and doses are not optimal and there is no awareness of these points even among experts involved in the treatment of this disease. Radiation treatment with higher radiation dose and longer radiation fields may improve the efficacy and success of this modality in esophageal cancer management and decrease the necessity of surgery. We demonstrated these assumptions in our study, and we think that our approach may cure more patients with esophageal cancer.

## Abbreviations

2D: Two-dimensional
3D: Three-dimensional
5-FU: 5-Fluorouracil
CDDP: Cisplatin
CTCAE: National Cancer Institute’s Common Terminology Criteria for Adverse Events
CTV: Clinical target volume
Def-CRT: Definitive concomitant chemoradiotherapy
GTV: Gross tumor volume
LR: Locoregional recurrence
PET-CT: Fluorodeoxyglucose positron emission tomography
Pre-CRT: Preoperative concomitant chemoradiotherapy
PTV: Planning target volume
RTOG: Radiation therapy oncology group
